# SMG1 Acts as a Novel Potential Tumor Suppressor with Epigenetic Inactivation in Acute Myeloid Leukemia

**DOI:** 10.3390/ijms150917065

**Published:** 2014-09-25

**Authors:** Yahui Du, Fei Lu, Peng Li, Jingjing Ye, Min Ji, Daoxin Ma, Chunyan Ji

**Affiliations:** Department of Hematology, Qilu Hospital, Shandong University, Jinan 250012, China; E-Mails: fengyinhuijing@126.com (Y.D.); lufei1985222@163.com (F.L.); pengli85@163.com (P.L.); jingjing_ye@163.com (J.Y.); jerrie981@163.com (M.J.); daoxinma@sdu.edu.cn (D.M.)

**Keywords:** acute myeloid leukemia (AML), decitabine, SMG1, mTOR

## Abstract

Suppressor with morphogenetic effect on genitalia family member (SMG1) belongs to a family of phosphoinositide 3-kinase-related kinases and is the main kinase involved in nonsense-mediated mRNA decay. Recently, *SMG1* was suggested as a novel potential tumor suppressor gene, particularly in hypoxic tumors. To investigate the function of *SMG1* in acute myeloid leukemia (AML), we performed methylation-specific polymerase chain reaction and found that *SMG1* was hypermethylated in the promoter region. *SMG1* hypermethylation was found in 66% (33/50) of AML samples compared with none (0/14) of the normal controls. SMG1 mRNA was down-regulated in AML patients with hypermethylation status whereas it was readily expressed in patients without methylation. Moreover, treatment of AML cells with demethylating agent 5-aza-2'-deoxycytidine (decitabine) inhibited AML cell growth and induced apoptosis by reversing *SMG1* methylation status and restoring SMG1 expression. On the other hand, knockdown of SMG1 by RNA interference inhibited apoptosis. We also found that mTOR expression level was negatively correlated to SMG1 expression in AML patients which indicated that SMG1 and mTOR maybe act antagonistically to regulate AML cell growth. In conclusion, our results indicate that *SMG1* acts as a potential tumor suppressor with epigenetic regulation in AML.

## 1. Introduction

Acute myeloid leukemia (AML) is a clonal disorder of hematopoiesis characterized by the uncontrolled proliferation and accumulation of immature and dysfunctional hematopoietic progenitors. Cytotoxic chemotherapy has been widely used as the main approach for AML treatment.

Recent studies have proved that, not only the successive accumulation of genetic alterations in oncogenes and tumor suppressor genes, but also the epigenetic alterations contribute to carcinogenesis [1]. The most extensively studied epigenetic mechanism is the methylation of the fifth carbon of a cytosine nucleotide. Alterations of the methylation status in oncogenes and tumor suppressor genes, which could affect the mRNAs and proteins expression levels, result in uncontrolled cell growth finally. Because epigenetic alterations are thought to be reversible, epigenetic drugs offer great promise for treatment of cancer.

5-Aza-2'-deoxycytidine (decitabine, DAC), as a kind of DNA methyltransferase inhibitor, is the most widely used epigenetic modulator to date. Recently, many studies have come to a conclusion that DAC offers a promising alternative therapeutic option for AML patients who are not candidates for standard remission induction chemotherapy [2] and DAC has activity in all phases of AML treatment [3]. Currently, the potential roles of DAC in the treatment of AML are being explored in numerous clinical trials.

In our study, we found that *SMG1* (suppressor with morphogenetic effect on genitalia family member) was hypermethylated in the promoter region in AML. SMG1 is a well known member of phosphoinositide 3-kinase-related kinases (PIKK) family. It is mainly involved in nonsense-mediated mRNA decay (NMD), which is the process of eliminating mRNAs that contain premature termination codons to prevent the accumulation of truncated proteins [4]. However, up to date *SMG1* functional mutations, deletions, or reduced expression in human cancer is rarely studied. Recently, *SMG1* was suggested to be a potential tumor suppressor [5] and could be down-regulated due to promoter hypermethylation in human papillomavirus (HPV)-positive head and neck squamous cell carcinoma [6]. However, there is no data showing the relation between *SMG1* promoter methylation status and its expression level in AML.

Cristina *et al.* reported that SMG1 and mammalian target of rapamycin complex 1 (mTORC1) act antagonistically to regulate response to injury and growth in planarians and their study indicated that SMG1 is likely to be a potential human tumor suppressor gene product [5]. Mammalian target of rapamycin (mTOR) signaling pathway regulates cell growth and proliferation and is essential for the process of protein synthesis, which is consistent with that many human genetic defects and tumors associating with mTOR up-regulation manifest as uncontrolled cell growth [7]. As a result mTOR signaling is currently the most targeted signaling pathway in drug development for the treatment of cancers. Many human tumor suppressors negatively regulate mTOR signaling. Although it has been proven that mTOR signaling pathway is over-activated in AML, the relation between SMG1 and mTOR remains unknown in AML so far.

In our study, we found that SMG1 expression level was negatively correlated with its methylation status and mTOR expression level respectively which indicated that SMG1 and mTOR may act antagonistically to regulate AML cell growth. In conclusion, our results indicate that *SMG1* acts as a potential tumor suppressor with epigenetic regulation and highlights a new approach for the demethylating treatment of DAC in AML.

## 2. Results

### 2.1. SMG1 Was Down-Regulated in Acute Myeloid Leukemia (AML) Patient Samples

**Figure 1:**
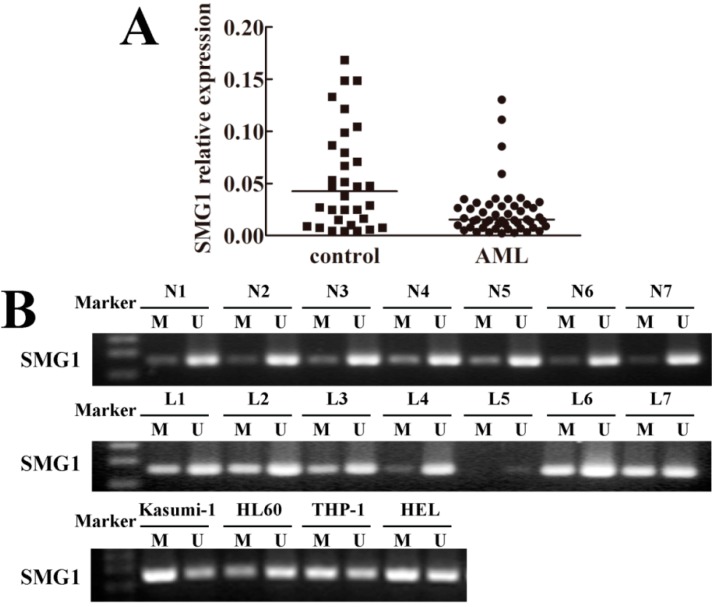
Epigenetic silencing of *SMG1* in acute myeloid leukemia (AML) patient samples and cell lines. (**A**) Down-regulation of SMG1 confirmed by quantitative RT-PCR (*p* < 0.05); (**B**) Representative Methylation-Specific Polymerase Chain Reaction (MSP) results of the *SMG1* methylation status in normal controls, AML samples and AML cell lines, respectively. *SMG1* was unmethylated in normal controls, but frequently hypermethylated in AML samples and cell lines. M, methylated product; U, unmethylated product; N, normal control; L, acute myeloid leukemia.

We performed quantitative Real-Time Polymerase Chain Reaction (RT-PCR) to detect SMG1 mRNA expression in bone marrow samples from 50 AML patients and 32 normal controls. The results showed that SMG1 was down-regulated in AML, but was readily detected in the controls as shown in Figure 1A. These results suggested aberrant gene silencing of *SMG1* in AML.

### 2.2. Hypermethylation Status of SMG1 Gene Was Associated with Transcriptional Down-Regulation

In order to investigate whether the promoter hypermethylation of *SMG1* gene results in the reduced SMG1 mRNA expression, we performed Methylation-Specific Polymerase Chain Reaction (MSP) to analyze the methylation status of *SMG1* gene in AML. The *SMG1* methylation status was detected in 50 samples of AML patients and 14 samples of normal controls. We found *SMG1* hypermethylation in 33 out of 50 (66%) AML samples and no *SMG1* hypermethylation (0/14) in the controls. *SMG1* was also methylated by varying degrees in four AML cell lines (Kasumi-1, HL60, THP-1, HEL) in our study. The representative results are shown in Figure 1B and the detailed results are represented in Figure S1. These results suggested that hypermethylation in the promoter region of *SMG1* may be responsible for transcriptional down-regulation.

### 2.3. 5-Aza-2'-deoxycytidine (Decitabine, DAC) Decreased Methylation Degree of SMG1 and Restored SMG1 Expression

**Figure 2:**
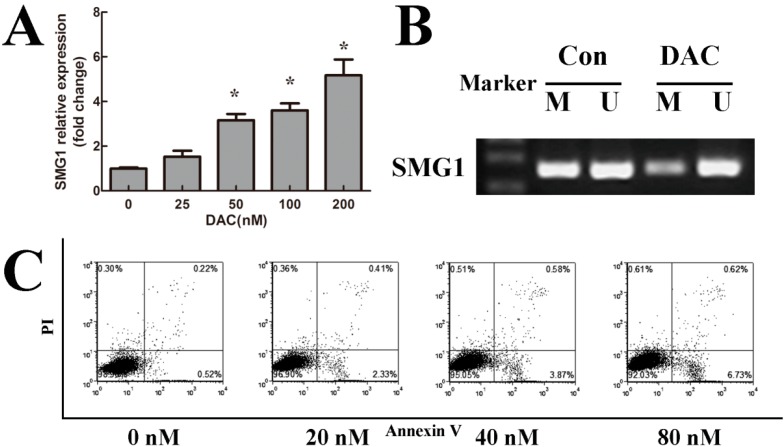
DAC treatment induced AML HEL cell apoptosis via reversing *SMG1* methylation status and restoring SMG1 mRNA expression. (**A**) DAC restored SMG1 mRNA expression in HEL cells with a dose-dependent effect confirmed by quantitative RT-PCR. * *p* < 0.05 compared with the 0 nM DAC treatment group; (**B**) Hypermethylation status of *SMG1* in HEL cell reversed by DAC. M, methylated product; U, unmethylated product; Con, DMSO only; DAC, decitabine treatment; (**C**) DAC treatment induced HEL cell apoptosis with dose-dependent effect analyzed by flow cytometry. DAC treatment caused a significant increase in early apoptotic cells and late apoptotic cells compared with that of the untreated group.

To further test the hypothesis that the DNA hypermethylation in the promoter region of *SMG1* caused the reduced expression of SMG1, firstly we performed MSP to test the demethylating function of DAC. As shown in Figure 2B, HEL cells treated with DAC showed reversed methylation status compared with control group .According to this result, we confirmed the demethylating effect of DAC and demonstrated that DAC regulated SMG1 expression by changing the methylation status of *SMG1* gene.

Then, we performed quantitative RT-PCR to detect the SMG1 expression in DAC treatment group and control group in HEL cells. As shown in Figure 2A, HEL cells treated with DAC were induced to express SMG1. The ratio of SMG1 to β-actin transcripts was increased several folds in the DAC-treated group compared with that in the control group. These results strongly indicated that hypermethylation in the promoter region of *SMG1* gene was responsible for the decreased expression of SMG1 in AML.

### 2.4. DAC Inhibited Cell Growth and Induced Apoptosis

To determine whether the effect of DAC was related to apoptosis, we performed flow cytometry and found that DAC treatment caused a significant increase in early apoptotic cells and late apoptotic cells compared with that of the control group (Figure 2C). These results suggested that DAC negatively regulated AML cell growth and we speculated that the demethylation of *SMG1* may play a role in the above functional effect of DAC in AML cells.

### 2.5. Knockdown of SMG1 Inhibited Apoptosis

**Figure 3:**
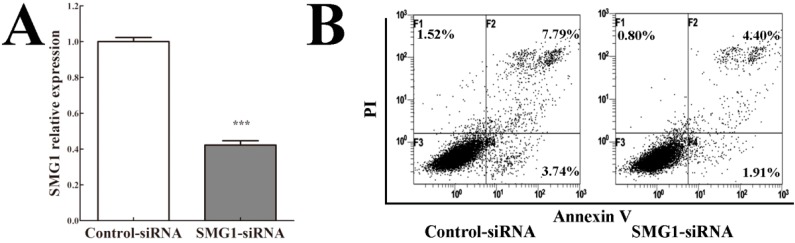
Effect of *SMG1* knockdown on apoptosis in AML HEL cells. (**A**) SMG1 mRNA-targeting siRNA and a control-siRNA were transfected in SMG1-expressing HEL cells. The efficiency of *SMG1* knockdown was examined by quantitative RT-PCR. *** *p* < 0.001 compared with the control-siRNA group; (**B**) *SMG1* knockdown affect HEL cells apoptosis confirmed by flow cytometry.

To evaluate the functional significance of *SMG1* in AML, we knocked down *SMG1* in HEL cells by siRNA. Quantitative RT-PCR results showed that SMG1expression was significantly decreased by SMG1-siRNA (Figure 3A). Then, we performed flow cytometry to examine the effect of *SMG1* knockdown on apoptosis. The results showed that both early apoptotic cells and late apoptotic cells among SMG1–siRNA-transfected HEL cells decreased compared with those among control–siRNA-transfected HEL cells (Figure 3B). These data suggested that AML cell growth required normal SMG1 level to place a brake on proliferation and indicated that *SMG1* acted as a potential tumor suppressor gene in AML.

### 2.6. mTOR Expression Level Was Negatively Correlated with SMG1 in AML

**Figure 4:**
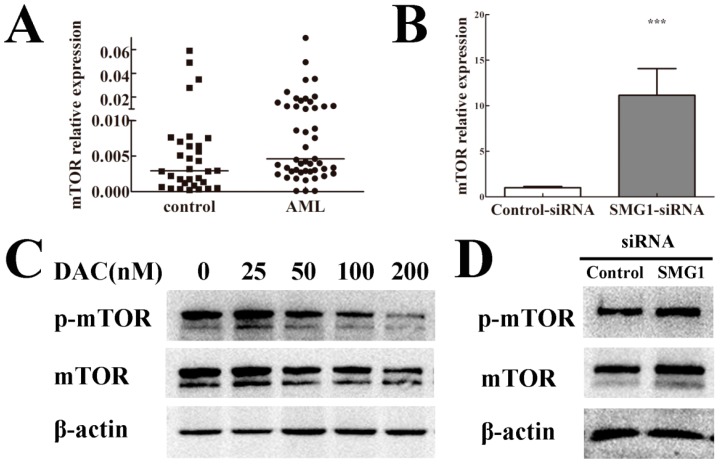
mTOR maybe act antagonistically with SMG1 to regulate cell growth. (**A**) Over-expression of mTOR in AML compared with controls confirmed by quantitative RT-PCR (*p* < 0.05); (**B**) Effect of *SMG1* knockdown on mTOR mRNA expression in HEL cells. mTOR expression was increased in SMG1–siRNA-transfected group compared with the control–siRNA group confirmed by quantitative RT-PCR (*** *p* < 0.001); (**C**) DAC treatment decreased both mTOR and *p*-mTOR protein expression with dose-dependent effect confirmed by western blotting analysis; (**D**) mTOR and *p*-mTOR protein expression was increased in SMG1–siRNA-transfected group compared with the control–siRNA group confirmed by western blotting analysis.

It is known that mTOR signaling is essential for cell growth and proliferation in all eukaryotes analyzed so far. Many negative regulators of mTOR signaling are known human tumor suppressors. To determine whether the expression level of mTOR was related to SMG1, firstly we performed quantitative RT-PCR and found that mTOR was over-expressed in AML samples compared with that in normal controls (Figure 4A). We also found that mTOR was expressed at lower levels in the DAC treated group compared with the control group confirmed by western blotting analysis (Figure 4C) while both its mRNA and protein expression level increased in SMG1-siRNA-transfected group compared to that in the control–siRNA-transfected group confirmed by quantitative RT-PCR analysis and western blotting analysis (Figure 4B,D). Taken together, these results showed that mTOR expression level was negatively correlated with the SMG1 expression in AML which indicated that SMG1 and mTOR maybe act antagonistically to regulate cell growth in AML.

## 3. Discussion

Cancer is a disease that results from the successive accumulation of genetic and epigenetic alterations. Many studies have proved the hypothesis that aberrant epigenetic regulations, resulting from an abnormal DNA methylation and histone modification status, played an important role in AML pathogenesis [8,9]. Epigenetic silencing of many novel potential tumor suppressor genes has been reported to contribute to AML and other kind of cancers [10]. Recently, *SMG1* was suggested as a novel tumor suppressor gene, though its role as a NMD effector has been well known. Here, we identified that the promoter region of *SMG1* gene showed a hypermethylation status in AML. To our knowledge, it was the first time that the epigenetic regulation of SMG1 expression and its function in AML was studied.

In this study, we found that the hypermethylation status of *SMG1* was frequent and cancer specific in AML. Our results suggest that *SMG1* methylation is associated with AML pathogenesis. In accordance with our study, SMG1 was down-regulated due to its promoter hypermethylation and its over-expression protected HPV-positive cells from radiation in head and neck squamous cell carcinoma [6]. We also found that the demethylating agent DAC decreased the methylation degree of *SMG1* and restored its mRNA expression, which suggested that the CpG island methylation was the predominant regulatory mechanism of SMG1 inactivation in AML.

As we all know, DNA methylation is established by DNA methyltransferases (DNMTs). DNMT3A, a major player in the *de novo* DNA methylation at CpG sites, is one of the most frequently mutated genes in AML [11]. Despite as an independent marker of adverse prognosis in AML [12], the biochemical effect of DNMT3A mutations on DNA methylation has not been definitively delineated. A group reported that global levels of DNA methylation did not differ between DNMT3A wild-type and mutant patients [13]. The mechanism of *SMG1* methylation regulation has been barely studied so far. In the future, the relationship between *SMG1* hypermethylation and DNMT3A mutations requires further study. 

Despite being involved in NMD, SMG1 has many other biological functions, such as maintaining telomere integrity, protecting against TNF-α-induced apoptosis, regulating lifespan and oxidative stress resistance, having an essential role in embryogenesis and activating p53 and an important role in the DNA damage response network [14,15,16,17]. Cristina *et al.* found that SMG1 was required for the tight control of stem cell proliferation and differentiation caused by injury or nutrient status in planarian flatworms [5]. Knockdown of *SMG1* in planarian flatworms leads to lethal outgrowths which display several hallmarks of human cancers, which is similar with the knockdown of known human tumor suppressors such as PTEN or p53. These studies suggested that *SMG1* might have a potential tumor suppressor role in human cancer. It will be very interesting to investigate this new role in AML.

Thus, the tumor suppressor role of *SMG1* was investigated in AML. We found that mRNA expression level of SMG1 was negatively associated with the hypermethylation status, which could be reversed by DAC treatment. DAC also restored mRNA expression of SMG1 and induced AML cells apoptosis. It is plausible to assume that DAC has an effect on the cell growth and proliferation via regulating SMG1. On the other hand, knockdown of *SMG1* by siRNA inhibited AML cell apoptosis. These results indicate that *SMG1* functions as a tumor suppressor in AML.

It has been widely accepted that aberrant activation of the PIKK/AKT/mTOR pathway promoted AML cell proliferation and survival [18]. mTOR is a central element of PIKK/AKT/mTOR pathway and a key kinase activating downstream of PIKK/AKT. Many studies underway are focused on the development of inhibitors of this signaling pathway [19]. Though SMG1 and mTOR both belong to the PIKK family, the interactions between them are not fully understood and insights into mTOR and SMG1 regulation are pertinent. In our study, we found that mTOR expression was negatively correlated with that of SMG1, which raised the possibility that SMG1 has either a direct or indirect interaction with mTOR signaling. Cristina *et al.* also described almost polar opposite roles for mTOR and SMG1 in planarian flatworms. The study of Robert *et al.* suggested that the mTOR pathway may be targeted and inhibited tumor suppressor elements with regulatory effects on mRNA translation [20]. Future genetic and biochemical study will help elucidate the relationship between SMG1 and mTOR signaling.

## 4. Experimental Section

### 4.1. Patients and Ethics Statement

A total of 82 subjects were recruited for this study, including 50 AML patients and 32 healthy volunteers at the Qilu Hospital of Shandong University (Jinan, China). Our research was approved by the Medical Ethical Committee of Qilu Hospital of Shandong University (Register ID No.: KYLL-2013-044; 21 February 2013). A written informed consent document has been obtained from each participant including the guardians on the behalf of the minors/children participants involved in our study. Mononuclear cells from bone marrow aspirates were isolated by density-gradient centrifugation with the use of Ficoll-Paque Plus (Ficoll, Pharmacia LKB Biotechnology, Piscataway, NY, USA). Detailed clinical information for AML patients is summarized in Table 1.

**Table 1:** Characteristics of the 50 AML patients.

Variables	AML Patients
Sex (male/female)	25/25
Age: median years (range)	45.50 (14–83)
WBC count (10^9^/L) median: (range)	37.28 (0.77–254)
Hemoglobin (g/dL) median: (range)	74.60 (45–117)
Platelet count (10^9^/L) median: (range)	52.22 (4–263)
**FAB Classification**	**Number of Patients**
M0	0
M1	0
M2	6
M3	14
M4	6
M5	24
M6	0

Abbreviations: FAB, French–American–British classification; WBC, white blood cells; M0, minimally differentiated AML; M1, AML without maturation; M2, AML with maturation; M3, acute promyelocytic leukemia; M4, acute myelomonocytic leukemia; M5, acute monocytic leukemia; M6, erythroleukemia.

### 4.2. Cell Culture

The human AML cell lines Kasumi-1, HL60, THP-1 and HEL were purchased from Institute of Hematology & Blood Diseases Hospital, Chinese Academy of Medical Science & Peking Union Medical College (Tianjin, China). Cells were cultured in complete medium (RPMI-1640 supplemented with 10% fetal bovine serum, 100 U/mL penicillin G, 100 µg/mL streptomycin, and 2 mM l-glutamine), at 37 °C in humidified air containing 5% carbon dioxide air atmosphere and were routinely subcultured every 2–3 days.

### 4.3. DNA Extraction and Methylation-Specific Polymerase Chain Reaction (MSP)

Genomic DNA from patient samples was isolated by DNA Extraction Kit (TIANGEN, Beijing, China) and chemically modified by using an EZ DNA Methylation-Gold Kit (Zymo Research, Orange, CA, USA) according to the manufacturer’s instructions. The methylation status of *SMG1* promoter region was determined by methylation-specific polymerase chain reaction (MSP). The *SMG1* promoter region is located approximately 1.3 kb up stream and up to 200 bp downstream of the transcription initiation site, which has a GC content up to 64.6% [6]. Primers distinguishing unmethylated (U) and methylated (M) alleles were designed to amplify the sequence:

*SMG1* M-forward primer sequence: 5'-GCGTACGTGAATTTAAGGGTAC-3';
*SMG1* M-reverse primer sequence: 5'-AACAAAAAATCTCCACTACTACGAC-3';
*SMG1* U-forward primer sequence: 5'-GGTGTATGTGAATTTAAGGGTATGT-3';
*SMG1* U-reverse primer sequence: 5'-AACAAAAAATCTCCACTACTACAAC-3'.

### 4.4. Demethylating Agent DAC Treatment

Demethylation was performed by using DAC. AML cells were seeded at a density of 1 × 10^6^ cells per mL in 10-cm dishes. After 24 h of culture, cells were treated with DAC for 2 days. Control cells were treated in parallel with DMSO. The medium was replaced every day. Cells were harvested and mRNA expression of SMG1 and mTOR were analyzed by quantitative RT-PCR.

### 4.5. SMG1 Knockdown and Transfection

The synthetic SMG1–siRNA and controls were purchased from Ribobio (Guangzhou Ribo Bio Co., Ltd., Guangzhou, China). Transfection of siRNA was performed with Lipofectamine 2000 reagent (Invitrogen, Carlsbad, CA, USA) in accordance with the manufacturer’s protocol. AML cell lines with SMG1 expression were transfected with SMG1–siRNA or control–siRNAs. Knockdown efficiency was evaluated at 48 h after transfection by quantitative RT-PCR. The siRNA with the highest knockdown efficiency was used for the following functional studies.

### 4.6. Quantitative Real-Time Polymerase Chain Reaction (RT-PCR) Analysis

Total RNA was extracted from all samples using TRIzol reagent (Invitrogen). To detect SMG1, mTOR expression, cDNA was synthesized from about 1 µg total mRNA using the M-MLV RTase cDNA Synthesis Kit (Takara, Dalian, China) according to the manufacturer’s instructions. Quantitative RT-PCR was conducted on an Applied Biosystems 7900HT System (ABI, Foster, CA, USA) with SYBR Green PCR Master Mix (Toyobo, Osaka, Japan). β-Actin was used to normalize expression levels of SMG1 and mTOR. Each sample was measured three times at least and fold changes in mRNA expression levels were calculated using the comparative threshold cycle method.

### 4.7. Apoptosis Assay

Apoptosis was detected using an Annexin V/FITC and PI Apoptosis Detection Kit (BestBio, Shanghai, China). Briefly, after treatment with DAC or transfection with SMG1–siRNA for 48 h, 2 × 10^5^ cells were harvested, resuspended in 100 µL flow cytometry binding buffer, and stained with 5 µL Annexin V/FITC followed by 1 µL PI. Cells were then incubated in the dark for 15 min at room temperature, and added 400 µL binding buffer. Cells were immediately measured by Galios flow cytometer (Beckman Coulter, CA, USA).

### 4.8. Western Blot Analysis

AML cells were lysed by Total Protein Extraction Kit (BestBio, Shanghai, China) and protein samples were stored at −80 °C. Proteins were separated by SDS-polyacrylamide gel electrophoresis and transferred to polyvinylidene difluoride membranes, which were blocked with 5% nonfat dry milk in Tris-buffered saline containing 0.05% Tween 20 (TBST) for 2 h at room temperature. The membranes were then incubated with the following specific antibodies: rabbit anti-mTOR or rabbit anti-*p*-mTOR (Cell Signaling Technology, Beverly, MA, USA) overnight at 4 °C. After washing with TBST for 3 times, membranes were incubated with secondary antibodies (goat peroxidase-conjugated anti-rabbit immunoglobulin; Santa Cruz Biotechnology, Dallas, TX, USA) for 1.5 h at room temperature. Protein bands visualized by FluorChem E Chemiluminescent Western Blot Imaging System (Cell Biosciences, Santa Clara, CA, USA).

### 4.9. Statistical Analysis

Data was collected from at least three experiments in duplicate. Statistical analysis was carried out using SPSS software (version 17.0; Chicago, IL, USA). Differences with *p*-values of less than 0.05 were considered statistically significant. The statistical comparisons between the controls and AML patients were made with the Mann-Whitney U nonparametric tests.

## 5. Conclusions

Taken together, our findings suggest that SMG1 acts as a novel functional tumor suppressor gene which was down-regulated by CpG island hypermethylation in AML. SMG1 methylation may serve as a potential epigenetic biomarker for early diagnosis of AML patients. SMG1 and mTOR may act antagonistically to regulate AML cell growth and proliferation. Our study highlights a new approach for the demethylating treatment of DAC in AML.

## Supplementary Materials

Supplementary Figure can be found at http://www.mdpi.com/1422-0067/15/9/17065/s1.

## Supplementary Files

Supplementary File 1
