# Supplementary material for: SMG1 Acts as a Novel Potential Tumor Suppressor with Epigenetic Inactivation in Acute Myeloid Leukemia
Source: Int J Mol Sci. 2014 Sep 25;15(9):17065–76. doi: 10.3390/ijms150917065 (PMC4200422; doi:10.3390/ijms150917065)

Supplementary Information

Figure S1. MSP results of the SMG1 methylation status in other 7 normal controls and 43 AML samples. SMG1 was unmethylated in normal controls, but frequently hypermethylated in AML patient samples and cell lines. M, methylated product; U, unmethylated product; N, normal control; L, acute myeloid leukemia.

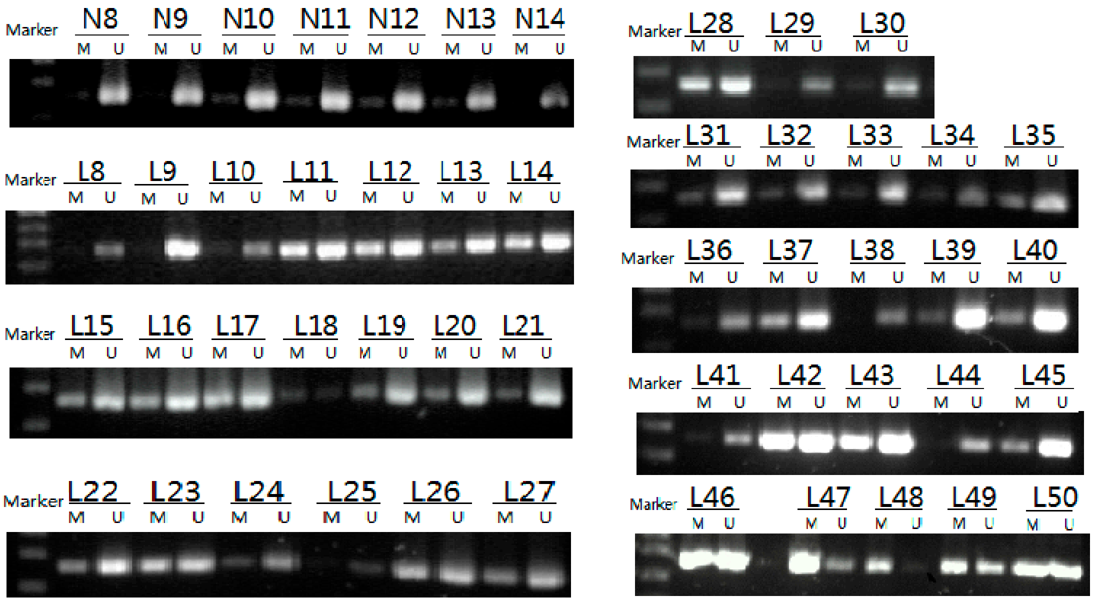

Supplement: Supplementary File 1 [file ijms-15-17065-s001.pdf]
